# Loss of Cytokine-STAT5 Signaling in the CNS and Pituitary Gland Alters Energy Balance and Leads to Obesity

**DOI:** 10.1371/journal.pone.0001639

**Published:** 2008-02-20

**Authors:** Ji-Yeon Lee, Heike Muenzberg, Oksana Gavrilova, Jacquelyn A. Reed, Darlene Berryman, Eneida C. Villanueva, Gwendolyn W. Louis, Gina M. Leinninger, Stefano Bertuzzi, Randy J. Seeley, Gertraud W. Robinson, Martin G. Myers, Lothar Hennighausen

**Affiliations:** 1 Laboratory of Genetics and Physiology, National Institute of Diabetes and Digestive and Kidney Diseases, National Institutes of Health, Bethesda, Maryland, United States of America; 2 Department of Medicine and Molecular and Integrative Physiology, University of Michigan, Ann Arbor, Michigan, United States of America; 3 Mouse Metabolic Core Facility, National Institute of Diabetes and Digestive and Kidney Diseases, National Institutes of Health, Bethesda, Maryland, United States of America; 4 University of Cincinnati, Cincinnati, Ohio, United States of America; 5 Ohio University, Athens, Ohio, United States of America; 6 Mammalian Development Section, National Institute of Neurological Disorders and Stroke, National Institutes of Health, Bethesda, Maryland, United States of America; University of Parma, Italy

## Abstract

Signal transducers and activators of transcription (STATs) are critical components of cytokine signaling pathways. STAT5A and STAT5B (STAT5), the most promiscuous members of this family, are highly expressed in specific populations of hypothalamic neurons in regions known to mediate the actions of cytokines in the regulation of energy balance. To test the hypothesis that STAT5 signaling is essential to energy homeostasis, we used Cre-mediated recombination to delete the *Stat5* locus in the CNS. Mutant males and females developed severe obesity with hyperphagia, impaired thermal regulation in response to cold, hyperleptinemia and insulin resistance. Furthermore, central administration of GM-CSF mediated the nuclear accumulation of STAT5 in hypothalamic neurons and reduced food intake in control but not in mutant mice. These results demonstrate that STAT5 mediates energy homeostasis in response to endogenous cytokines such as GM-CSF.

## Introduction

The central nervous system modulates feeding and energy expenditure to maintain energy balance and metabolism within a precise homeostatic window. Many of the neural circuits that underlie this regulation lie within the hypothalamus, where basomedial “satiety” centers, including the arcuate nucleus (Arc), relay homeostatic information to regions that regulate satiety and energy expenditure, such as the paraventricular hypothalamic nucleus (PVN) and brainstem [1]. The lateral hypothalamic area (LHA) conversely mediates a variety of appetite-promoting signals. Cytokines mediate many of the physiologic cues that activate neuronal signaling in these regions to regulate energy balance. Notably, ciliary neurotrophic factor (CNTF), the adipocytokine leptin, and granulocyte-macrophage colony-stimulating factor (GM-CSF) and IL-6 regulate food intake and body weight via the activation of endogenous hypothalamic cytokine receptors that mediate signaling via the Janus kinase 2 (JAK2) and signal transducer and activator of transcription (STAT) pathways. Mouse genetics has demonstrated that STAT3 [2], [3], [4], [5] is essential for the regulation of energy balance in response to leptin and other endogenous cytokines. Similarly, neural SOCS3, an inhibitor of JAK2 and a variety of STATs, blocks signaling by several cytokines and thereby increases feeding and body weight by attenuating cytokine signaling in the CNS [6]. Reducing leptin signaling stimulates food intake and weight gain [7]. Like leptin, central administration of the proinflammatory cytokine GM-CSF suppresses food intake and GM-CSF^−/−^ mice are heavier and more obese than control mice [8]. Unlike leptin and CNTF, which activate STAT3 more robustly than STAT5A/B (STAT5), GM-CSF preferentially activates STAT5. Indeed, GM-CSF-dependent proliferation of macrophages is dependent on the presence of STAT5A [9].

STAT5 is expressed in distinct neuronal populations in the Arc [10], [11], [12], including dopaminergic [12] and somatostatin neurons [13]. Based on the knowledge that STAT5 and the GM-CSF receptor are present in hypothalamic neurons, we asked whether STAT5 contributes to the regulation of energy homeostasis and whether the action of GM-CSF on the regulation of energy balance depends on its ability to increase STAT5 activity in the CNS. This hypothesis was tested through the deletion of the *Stat5* locus using the *Nestin-Cre* transgene, which is active throughout the CNS and the pituitary gland [14], [15]. Here we present the effect of loss of STAT5 in neurons and establish a connection between GM-CSF and STAT5 in controlling energy homeostasis.

## Results

### STAT5 expression in hypothalamic neurons and neuronal deletion of STAT5A/B

**Figure 1:**
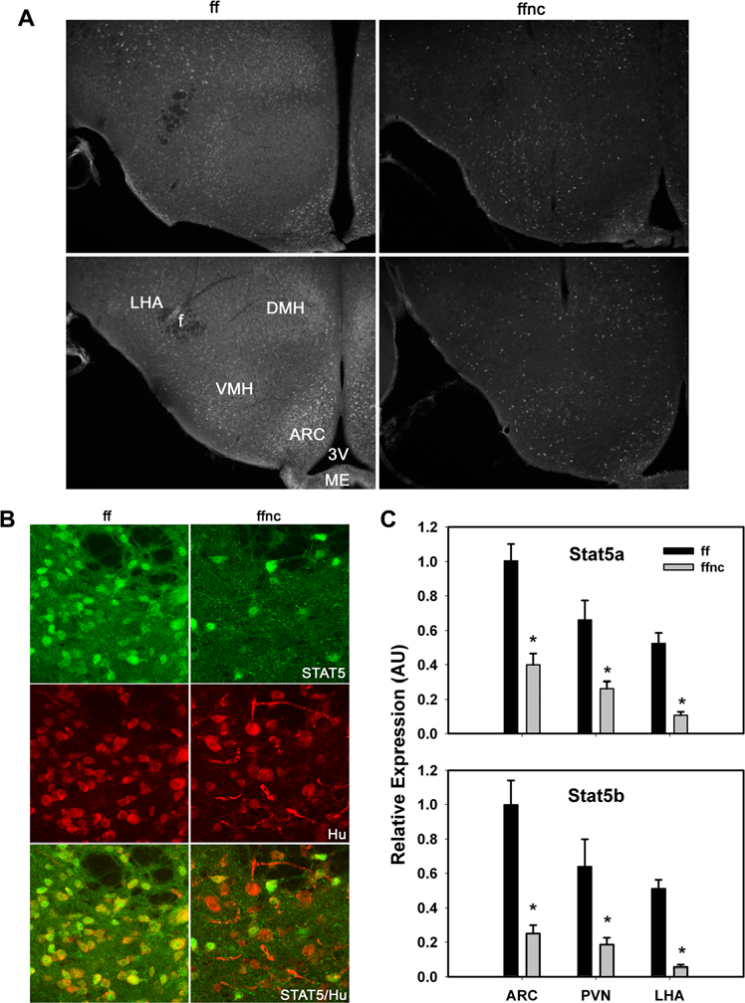
Reduced STAT5 expression in hypothalamic neurons in *Stat5^fl/fl; Nestin-Cre^* male mice. (A) Immunohistochemical analyses with αSTAT5 antibodies were performed on hypothalamic tissues from *Stat5^fl/fl^* control (ff, left panels) and *Stat5^fl/fl; Nestin-Cre^* (ffnc, right panels) mice. Note the prominent expression of STAT5 isoforms in discrete neurons and the dramatic reduction in STAT5-positive cells in ffnc mice. Upper panels show hypothalamic sections rostral to those shown in lower panels. 3V = third cerebral ventricle, ME = median eminence, f = fornix; all other abbreviations as defined in the text. (B) Immunostaining for STAT5 (green, top), the neural marker, Hu (red, middle) and merged images (bottom) in the LHA ff and ffnc animals. This demonstrates the co-localization/expression of STAT5 in neurons of ff animals and the absence of STAT5 from many Hu-positive neurons in ffnc mice. (C) Total RNA from microdissected hypothalamic subregions of ff and ffnc mice was subjected to semi-quantitative real-time PCR for STAT5A and STAT5B mRNA expression, confirming the reduction in the expression of both STAT5 isoforms in each region.

Immunohistochemical analysis using antisera reactive with both STAT5 isoforms demonstrated the prominent presence of STAT5 in discrete neuronal populations in a number of areas in the adult brain including basal ganglia, septum, cortex (not shown) and hypothalamus (Figure 1A). Particularly relevant to the phenotype described in this study, we detected STAT5 in a limited number of cells within areas of the hypothalamus known to regulate feeding and energy balance (Figure 1A), including the Arc, dorsomedial and ventromedial hypothalamic nuclei (DMH and VMH), and the LHA, suggesting a potential role for Stat5 in energy homeostasis.

Since *Stat5^-/-^* mice die perinatally [16], we used Cre-mediated recombination to delete the *Stat5* locus throughout the brain, including the hypothalamus. Mice carrying the *Stat5^fl/fl^* locus (in which the genes encoding STAT5A and STAT5B are flanked by loxP sites) [16] were bred with Nestin-Cre transgenic mice [14], [15] to produce Stat5*^fl/fl; Nestin-Cre^* mice and Stat5*^fl/fl^* controls. Immunofluorescent examination of STAT5 expression in the hypothalamus of these animals demonstrated overall decreased STAT5 immunoreactivity and the loss of detectable STAT5 protein from the majority of neurons in the hypothalamus (Figure 1A and 1B). Immunofluorescent staining for STAT5 and the neuron-specific marker Hu [17] revealed the extensive co-localization of STAT5 and Hu in control mice and the absence of STAT5 from most Hu-expressing neurons in Stat5*^fl/fl; Nestin-Cre^* mice (Figure 1B). We additionally assessed the loss of STAT5 isoforms from hypothalamic nuclei known to be central to the regulation of energy balance by semi-quantitative RT-PCR analysis of STAT5A and STAT5B mRNA from microdissected Arc, PVN, and LHA tissues of control and Stat5*^fl/fl; Nestin-Cre^* mice (Figure 1C). This analysis revealed the approximately 50–80% reduction in STAT5A mRNA and 75–90% reduction in STAT5B mRNA in all areas examined, with the most prominent reduction in the LHA (Figure 1C). These data are consistent with efficient excision of the *Stat5* locus from regions of the hypothalamus that regulate energy balance in the Stat5*^fl/fl; Nestin-Cre^* mice.

### Neuronal deletion of the *Stat5* locus results in obesity

**Figure 2:**
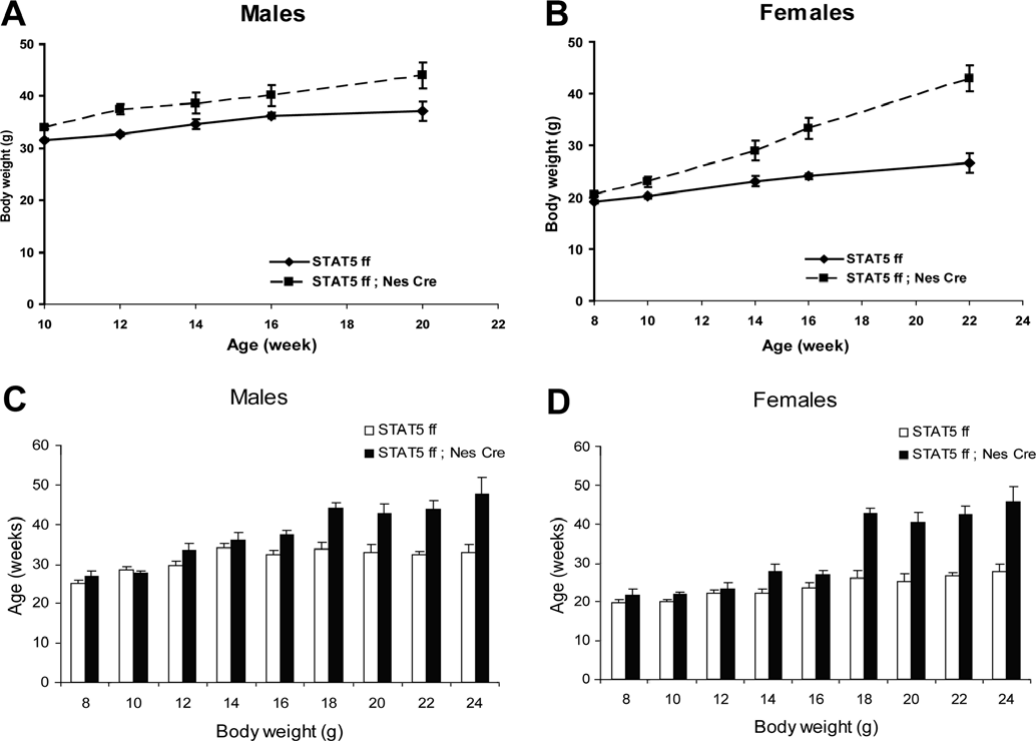
Increased body weight of *Stat5^fl/fl; Nestin-Cre^* mice. Body weight curves of males (A, *Stat5^fl/fl^*, n = 14; *Stat5^fl/fl; Nestin-Cre^*, n = 8) and females (B, *Stat5^fl/fl^, n = 5; Stat5^fl/fl; Nestin-Cre^* n = 6). Two Way Repeated Measured ANOVA test revealed that the effect of the genotype on body weight was significant in males (F(1,103) = 5.77, P = 0.024) and females (F9(1,36) = 64.6, P<0.001) (B). Panels C and D show body weights at various ages combined from several cohorts of mice. Values are mean±SEM, n = 5–37 per group. Two Way ANOVA analysis of variance for each age group shows a significant effect of genotype on body weight at all ages tested: 8 weeks−F(1,70) = 4.5, P = 0.037; 10 weeks−F(1,89) = 24.3, P<0.001; 12 weeks−F(1,99) = 6.4, P = 0.013; 14 weeks−F(1,80) = 8.98, P = 0.004; 16 weeks−F(1,87) = 14.9; P<0.001; 18 weeks−F(1,22) = 56.1, P<0.001; 20 weeks−F(1,13) = 37.1, P<0.001; 22 weeks−F(1,20) = 18.6, P<0.001. The effect of gender on body weight was significant at weeks 8 through 16 (with F values ranging from 24 to 87, P<0.001). The interaction between genotype and gender was detected at week 10 only (F(1,89) = 11,2, P = 0.01).

**Figure 3:**
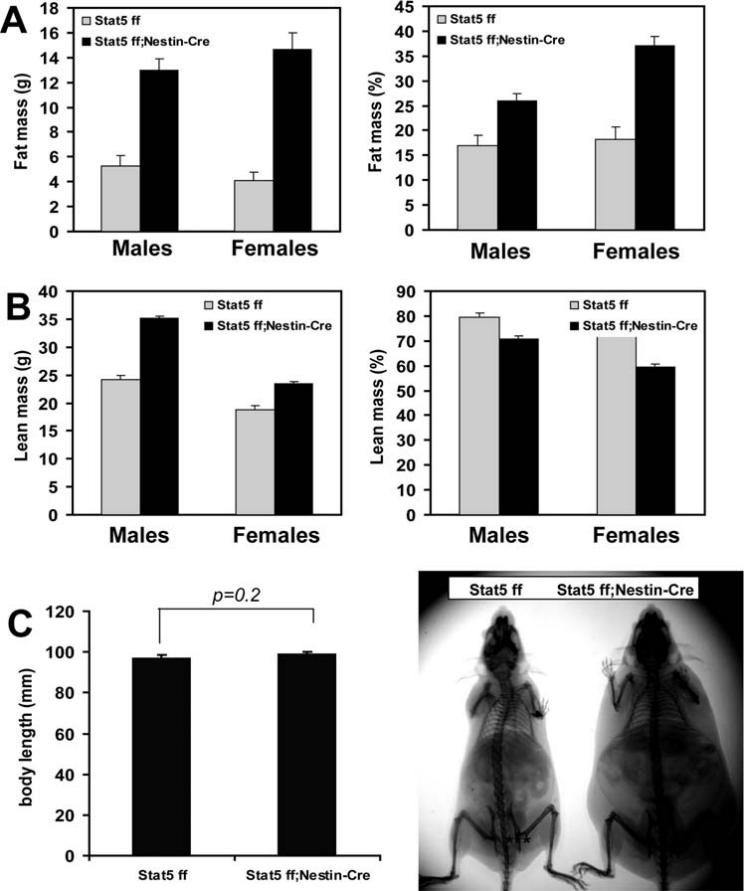
Increased body fat in *Stat5^fl/fl; Nestin-Cre^* mice. (A) Fat mass of 22 week-old males (n = 4/group) and females (n = 5/group). Left panel shows the amount of fat in grams. By Two Way ANOVA followed by Holm-Sidak test the effect of genotype was significant ( F(1,14) = 83.1, P<0.001). Right panel represents fat mass as a percentage of body weight (F(1,14) = 42.4, P<0.001 for the effect of genotype; F(1,14) = 8.0, P = 0.014 for the gender effect. (B) Lean mass of 22 week-old males and females in grams (left panel-( F(1,14) = 137, P<0.001 for the genotype effect; F(1,14) = 164, P<0.001 for the gender effect). Right panel presents lean mass as a percentage of total body weight (F(1,14) = 70.4, P<0.001, for the genotype effect, F(1,14) = 30.7 for the gender effect). Body weights were: *Stat5^fl/fl^* males 30.5±1.5, *Stat5^fl/fl; Nestin-Cre^* males–49.8±0.9 g; *Stat5^fl/fl^* females 25.3±1.3 g, *Stat5^fl/fl; Nestin-Cre^* females–39.4±1.5 g; F(1.14) = 143, P<0.001 for the genotype effect, F(1,14)+30 for the gender effect. (C) X-ray images of *Stat5^fl/fl; Nestin-Cre^* and *Stat5^fl/fl^* control male mice (right panel) showing similar size of bone length. There was no statistically significant difference in body length as shown in the graph (left panel).

Metabolic parameters and body weight of *Stat5^fl/fl; Nestin-Cre^* mice were monitored from 8 to 22 weeks of age. Both male and female mutant mice gained significantly more weight than control litter mates (Figure 2). Although 8 week-old mutant females were already significantly heavier than controls, differences in weight were more profound after 16 weeks of age. While the weight of control female mice increased only 10% between 12 and 22 weeks, mutants almost doubled their weight during this time interval (Figure 2B). By 26 weeks of age, *Stat5^fl/fl; Nestin-Cre^* mice weighed on the average 65% (males) and 60% (females) more than their control litter mates. The total amount of fat in *Stat5^fl/fl; Nestin-Cre^* male and female mice increased 2.5- and 3.5-fold, respectively (Figure 3A, left panel). Notably, the lean mass of mutant male and female mice increased by 40% and 20%, respectively (Figure 3B). Total body fat (expressed as percentage of body weight) in male and female *Stat5^fl/fl; Nestin-Cre^* mice was 150% and 200%, respectively of that measured in control mice (Figure 3A, right panel). Body size, as measured by snout-anus length, of mutant and control mice was similar (Figure 3C).

**Figure 4:**
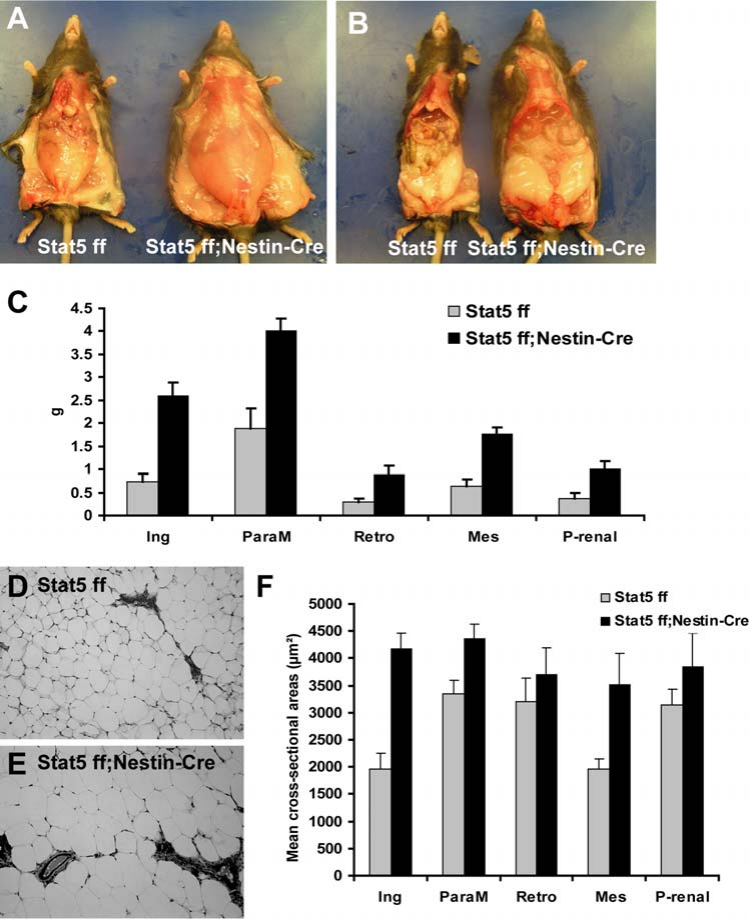
Analysis of fat depots and adipocyte size fat analysis from 22 week-old female mice. Subcutaneous (A) and visceral (B) fat were increased in *Stat5^fl/fl; Nestin-Cre^* mice, compared to *Stat5^fl/fl^* control mice. (C) Unpaired t-test was used for comparison of fat pad weights from *Stat5^fl/fl; Nestin-Cre^* and Stat5*^fl/fl^* control mice. Ing (inguinal, t(8) = 5.6, P<0.001), ParaM (parametrial, t(8) = 4.1, P = 0.004), Retro (retroperitoneal, t(8) = 2.9, P = 0.019), Mes (mesenteric, t(8) = 5.3, P<0.001)) and P-renal (perirenal, t(8) = 3.2, P<0.013). See figure 3 for body composition and body weight data. (D–E) Images of cross-sectional adipocytes from *Stat5^fl/fl^* (D) and *Stat5^fl/fl; Nestin-Cre^* mice (E). (F) Comparison of cell size distribution between two different genotypes for each fat pad. Values are mean cross-sectional area of each fat pad±SEM (n = 3–5 mice per group). Cross-sectional areas less than 100 µm^2^ were considered artifacts generated during image processing and were not used for statistical analysis. Adipocytes from *Stat5^fl/fl; Nestin-Cr^* mice were significantly larger in inguinal fat (t(8) = 5.3, p,0.001), parametrial fat (t(8) = 2.8, p = 0.02) and mesenteric fat (t(6) = 3.1, 0.02).

At 22 weeks of age, both subcutaneous (Figure 4A) and visceral (Figure 4B) fat mass were increased in *Stat5^fl/fl;Nestin-Cre^* mice compared to *Stat5^fl/fl^* control mice. Individual fat pads from control and mutant female mice were dissected and weighed to evaluate the distribution of fat mass. The weight of all 5 fat pads (inguinal, parametrial, retroperitoneal, mesenteric and perirenal) in mutant mice was at least doubled in mutant compared to control animals (Figure 4C). To determine whether increased adipose tissue mass was the result of an increased size of individual adipocytes, increased cell number or a combination of both, cell sizes were measured from paraffin embedded sections. In inguinal, parametrial and mesenteric fat pads, the mean cross-sectional areas of adipocytes in *Stat5^fl/fl; Nestin-Cre^* mice were significantly larger than this in *Stat5^fl/fl^* mice (Figure 4D–F), suggesting increased lipid storage contributes to the increased fat mass in these animals.

### STAT5 mutant mice have elevated leptin levels and display insulin resistance

**Figure 5:**
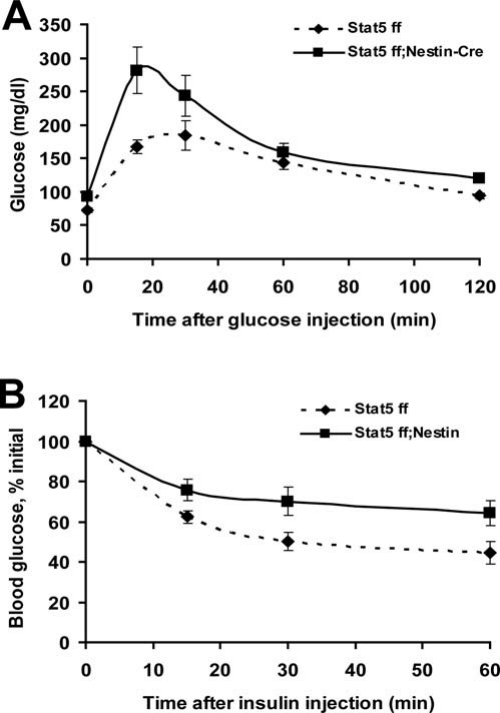
Glucose and insulin tolerance tests in 22 week-old *Stat5^fl/fl^* and *Stat5^fl/fl; Nestin-Cre^* mice. (A) Glucose tolerance tests were performed on fasted 22 week-old female mice after an i.p. injection of 2 g/kg BW glucose. Results are expressed as average blood glucose level±SEM of 5 females of each group. The effect of genotype was significant (Two Way Repeated Measured ANOVA with Holm-Sidak test: (F(1,24) = 6.6, p = 0.033. (B) Insulin tolerance test on 22 week-old females. Mice were fasted for 9 hours followed by the administration of 0.75U insulin per kg body weight. Results are expressed as average blood glucose level±SEM of 5 females of each group. The effect of genotype was significant (Two Way Repeated Measured ANOVA Holm-Sidak test: (F(1,24) = 7.3, p = 0.022). GTT and ITT were perform on the same set of *Stat5^fl/f^* and *Stat5^fl/fl; Nestin-Cre^* mice weighing mice 27.1±3.8 and 41.5±4.2 g, respectively.

Serum analysis of fed mice demonstrated elevated levels of TG, FFA, insulin and leptin in 22 week-old mice Stat5*^fl/fl^* ^; *Nestin-Cre*^ mice (Table 1). Notably, insulin and leptin levels were increased approximately 8-fold and 4.5-fold, respectively. Consistent with the increase in circulating insulin, Stat5*^fl/fl^* ^; *Nestin-Cr*e^ mice were glucose intolerant and insulin resistant (Figure 5). Thus, similar to many other mouse models of obesity, Stat5*^fl/fl^* ^; *Nestin-Cre*^ mice demonstrated some features of metabolic syndrome, including hyperlipidemia, hyperinsulinemia, insulin resistance and glucose intolerance. With the caveat that GH secretion in rodents is pulsatile, single measurements of GH levels suggests no differences between control and mutant mice. Normal IGF-1 levels (Table 1) and body lengths (Figure 3C) further argue for normal GH levels.

**Table 1:** Metabolic parameters in serum

	Stat5^fl/fl^	Stat5^fl/fl;Nestin-Cre^	*t*-test
TG (mg/dl)	63±8	200±63	t(8) = 2.1, p = 0.06
FFA (mM)	0.249±0.008	0.401±0.014	t(8) = 9.2, p<0.001
Insulin (ng/ml)	0.7±0.06	5.78±1.27	t(8) = 4.0, p = 0.004
Glucose (mg/dl)	181±4	185±10	ns
Leptin (ng/ml)	19.53±4.34	89.66±16.57	t(8) = 4.1, p = 0.003
IGF (ng/ml)	287.75±20	264.25±12.8	ns
Adiponectin µg/ml)	21.16±3.9	16.57±2.5	ns
GH (ng/ml)	9.25±0.85	9.25±0.25	ns

Blood was collected from the tail vein directly into non-EDTA-coated capillary tubes and centrifuged to separate the serum, which was used for assay. Values shown are the mean±SEM; four to five 5-months-old fed female mice were used for analysis. ns, not significant.

### STAT5 mutant mice have elevated food intake

**Figure 6:**
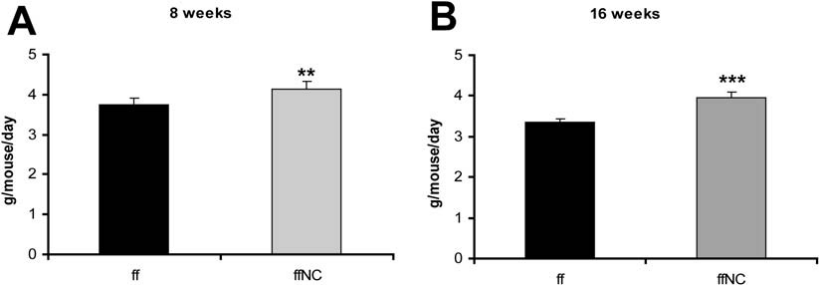
Food intake of *Stat5^fl/fl^* and *Stat5^fl/fl; Nestin-Cre^* mice was measured in a set of male mice (n = 6 per group) at the age of 8-weeks (A) and 16 weeks (B). Data show the total grams of food consumed per mouse per day. Mice were housed individually for all measurements. Values are mean±SEM. At 8 weeks of age, *Stat5^fl/f^* weighed 25.6±3.1 g and *Stat5^fl/fl; Nestin-Cre^* was 23.9+3.2 g; at 16 weeks body weights were 28.1±3.3 vs 29.2±3.7 respectively. By Two Way Repeated Measured ANOVA there were no effect of genotype on body weight in this cohort of male mice, however the effect of genotype on food intake was significantly different (F(1,10) = 5.7, p = 0.038. All pairwise multiple comparison (Holm-Sidak test) revealed that food intake was significantly different between two groups at 16 weeks of age (P = 0.0.005, t = 3.2), but not at 8 weeks of age.

To determine whether the increased body weight was associated with increased food consumption, we measured daily food intake every 4 weeks between the ages of 8 and 16 weeks. This analysis detected increased food intake in male but not in female *Stat5^fl/fl; Nestin-Cre^* mice during this period (Figure 6). At the age of 26 weeks both mutant males and females ate significantly more than controls per animal. When the data were normalized to lean mass, the difference remained significant in females (Table 2). To test whether changes in energy expenditure could also contribute to obesity in *Stat5^fl/fl; Nestin-Cre^* mice, we measured oxygen consumption and activity. At the age of 12 weeks oxygen consumption was comparable in control and mutant mice (data not shown). At 26 weeks of age, male *Stat5^fl/fl; Nestin-Cre^* were 60% heavier and were consuming more oxygen per mouse (Table 2). Oxygen consumption normalized to lean mass was significantly reduced in male *Stat5^fl/fl; Nestin-Cre^* compared to the control mice, but not in female mice (Table 2). In contrast, there was no difference in total activity, suggesting that changes in energy expenditure in males are likely attributable to reduced basal metabolic rates. Since increased food intake in males was observed prior to changes in energy expenditure, the data suggest that hyperphagia might be the primary cause of obesity in both male and female mice.

**Table 2:** Metabolic parameters of *Stat5^fl/fl^* and *Stat5^fl/fl^; ^Nestin-Cre^* mice

	Males			Females	Effect of strain	Effect of gender	Interaction
	Stat5*^fl/lf^*	Stat5*^fl/fl^*; Nestin-Cre	Stat5*^fl/lf^*	Stat5*^fl/fl^*; Nestin-Cre			
Body weight (g)	31.1±1.4	50.6±0.6*	26.8±1.1	32.9±0.7*	F(1,12) = 78.8, P<0.001	F(1,12) = 79.9, P<0.001	F(1,12) = 28.7, P<0.001
Lean mass (g)	23.9±0.8	35.5±0.4*	19.8±0.4	21.4±0.2	F(1,12) = 154.5, P<0.001	F(1,12) = 292.6, P<0.001	F(1,12) = 90.5, P<0.001
Fat mass (g)	6.2±1.2	12.8±0.5	5.7±1.4	9.4±0.1	F(1,12) = 22.2, P<0.001	NS	NS
Resting oxygen consumption (ml/mouse/h)	92.8±2.2	104.8±2.7*	80.4±1.5	89.2±3.6*	F(1,12) = 14.1, P = 0.003	F(1,12) = 25.9, P<0.001	NS
Resting oxygen consumption (ml/g^0.75 ^LM/h)	8.6±0.4	7.2±0.3*	8.6±0.1	9.0±0.4	NS	F(1,12) = 9.8, P = 0.009	F(1,12) = 8.9, P = 0.011
Resting respiratory exchange ratio (VCO2/VO2)	0.91±0.01	0.86±0.01	0.89±0.01	0.84±0.02	NS	NS	NS
Total oxygen consumption (ml/mouse/h)	107.2±2.7	129.7±3.8*	99.2±2.9	105.6±1.6	F(1,12) = 25.3, P<0.001	F(1,12) = 31.3, P<0.001	F(1,12) = 7.9, P = 0.016
Total oxygen consumption (ml/g^0.75 ^LM/h)	10.0±0.4	8.9±0.3*	10.5±0.3	10.6±0.1	NS	F(1,12) = 15.4, P = 0.002	NS
Total respiratory exchange ratio (VCO2/VO2)	0.91±0.01	0.89±0.01	0.90±0.01	0.85±0.03*	F(1,12) = 5.2, P = 0.041	NS	NS
Total activity at 23 °C (beam brake/min)	191.7±15.8	183.8±11.3	227.5±27	155.1±44.2	NS	NS	NS
Food intake (g/mouse/day)	2.9±0.3	4.3±0.2*	2.5±0.2	3.3±0.1*	F(1,12) = 28.3, P<0.001	F(1,12) = 11.5, P = 0.005	NS
Food intake (g/g^0.75^ LM/day)	0.266±0.025	0.293±0.010	0.263±0.0.017	0.330±0.009*	F(1,12) = 7.6, P<0.018	NS	NS

Oxygen consumption and CO_2_ production were measure at room temperature (23°C) at the age of 26 weeks.

Values shown are the mean±SEM; Four 6 month old fed mice were used for analysis. Statistical significance is from two-way ANOVA, followed by Holm-Sidak test (*indicate P<0.05 for comparison of strain effect within each gender).

### STAT5-mutant mice have reduced cold tolerance

**Figure 7:**
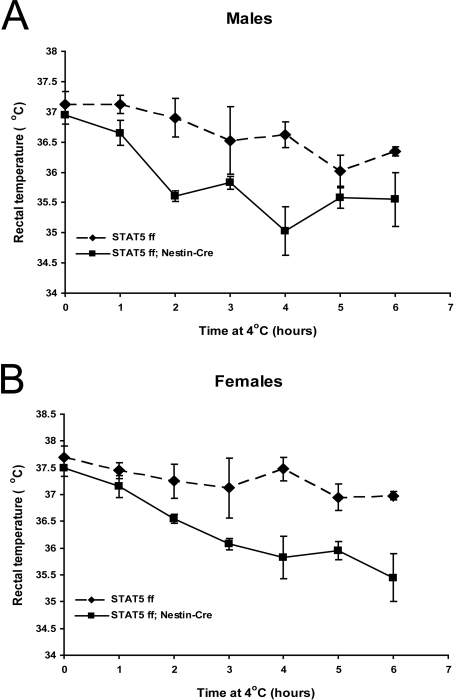
Stat5^fl/fl; Nestin-Cre^ mice are defective in cold-induced thermogenesis. 26 week-old Stat5*^fl/fl^* and Stat5*^fl/fl^* ^; Nestin-Cre^ males (A) and females (B) were housed at 4°C for 6 hours and rectal temperature was measured at 60-min intervals. The reduced cold tolerance in both males and females was statistically significant. N = 4/group. Statistical analysis was done using ‘Two Way Repeated Measures ANOVA’ followed by the ‘Holm-Sidak test’. The effect of genotype was significant in both females (F(1, 36) = 12.4, *P* = 0.013) and males (18.2, *P* = 0.005). The effect of length of cold exposure was also significant in females (*F*(6,36) = 9.5, *P*<0.001) and males (*F*(6,36) = 6.9, *P*<0.001

The CNS pathways that regulate food intake and energy balance overlap at many points with the systems that regulate body temperature. For example, STAT3 and cytokines such as leptin each contribute to regulate energy balance and the ability to maintain body temperature [3], [18]. We thus examined whether neuronal STAT5 similarly contributes to cold tolerance. *Stat5^fl/fl; Nestin-Cre^* and control female mice had body weights of 36.2 g and 25.6g, respectively. Mutant and control males had body weights of 50.3 g and 31.8 g, respectively. 26 week-old mice were housed at 4°C for 6 hours and body temperatures were measured every hour. While the body temperature of littermate controls dropped ∼0.7°C over the 6 hour period, the body temperature of mutant males decreased by ∼1.4°C (Figure 7A) and that of females by 2.0°C (Figure 7B). These data establish that neuronal STAT5 contributes to the regulation of body temperature, as well as to the control of food intake and body adiposity.

### Arcuate nucleus gene expression in STAT5-mutant mice

**Figure 8:**
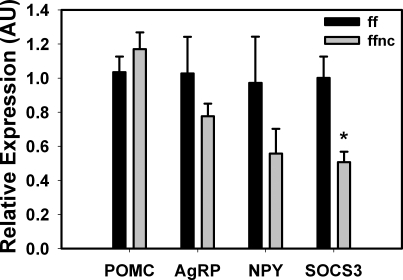
Expression of neuropeptide and regulatory genes in the Arc of Stat5*^fl/fl^* control (ff) and Stat5*^fl/fl^* ^; Nestin-Cre^ (ffnc) female mice. Total RNA prepared from microdissected Arc tissue from ff and ffnc mice was subjected to semi-quantitative real-time PCR for POMC, AgRP, NPY and SOCS3 mRNA expression. n = 9–10; * *t*(17) = 3.7, p = 0.002 by students t test, all other comparisons, p = NS.

In order to determine the potential dysregulation of Arc gene expression in *Stat5^fl/fl; Nestin-Cre^* mice, we microdissected the Arc of control and *Stat5^fl/fl; Nestin-Cre^* animals and analyzed the expression of the neuropeptides POMC, AgRP, and NPY, and the obesogenic JAK/STAT inhibitor SOCS3 by semi-quantitative qPCR (Figure 8). This analysis demonstrated no significant difference in neuropeptide gene expression in the Arc of *Stat5^fl/fl; Nestin-Cre^* animals but revealed decreased SOCS3 mRNA expression in the *Stat5^fl/fl; Nestin-Cre^* mice. This reduction in Arc SOCS3 mRNA expression in the STAT5 mutant mice is consistent with results from other tissues in which deletion of STAT5 reduces the baseline expression of SOCS3 [19], and demonstrates the importance of STAT5 in the regulation of *Socs3* gene expression in the hypothalamus as well as peripheral tissues. These data furthermore suggest that the regulation of energy balance by CNS STAT5-mediated transcriptional control is mediated by mRNAs other than those encoded by these Arc genes.

### STAT5 mediates GM-CSF actions in the CNS

**Figure 9:**
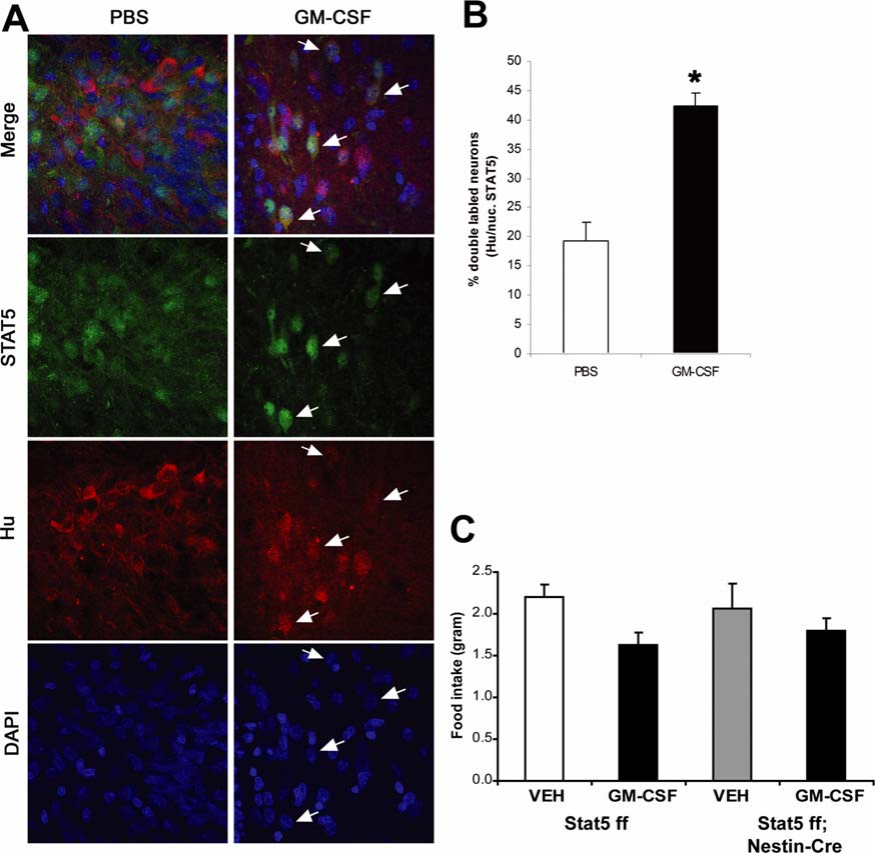
GM-CSF induces STAT5 activity in the hypothalamus and *Stat5^fl/fl; Nestin-Cre^* male mice do not respond to GM-CSF-induced reduction of food intake. (A) Rats were treated with vehicle (left panels) or GM-CSF (right panels) i3vt for 60 minutes and the presence of nuclear STAT5 was analyzed by counting STAT5-positive (green) nuclei of Hu-positive (red) neurons following immunofluorescent analysis with αSTAT5 antibodies. Dapi staining to reveal nuclei is shown in blue. Representative images from the LHA are shown, along with percent of neurons with nuclear STAT5 localization. (B) graph of double labeled cells; n = 4; *p<0.01 vs vehicle by students t test. (C) Recombinant mouse GM-CSF was injected into the third ventricular region of 8 week-old *Stat5^fl/fl; Nestin-Cre^* mice and *Stat5^fl/fl^* control littermates and food intake was measured after 4 hrs (N = 16 for Stat5 ff vehicle (body weight 29.8±0.9) and Stat5 ff GM-CSF ((body weight 29.4±0.9), n = 6–7 for Stat5 ff; Nestin-Cre (body weight 29.7±1.7) vehicle and Stat5 ff; Nestin-Cre GM-CSF(body weight 29.7±1.8)) . Two-way ANOVA tests followed by the ‘Holm-Sidak test’ revealed significant effect of GM-CSF on food intake (F(1,40) = 4.6, P = 0.039 showed a significant reduction of food intake with control mice and no significant reduction was observed with mutant mice. The p value for the drug effect was 0.039. Using ‘All pairwise multiple comparison procedures’ the p value for drug effect within wild type was 0.008. No significant effect was observed in the mutant group (p 0.435).

Central administration of GM-CSF to rats and mice decreases food intake and body weight [8]. Since STAT5 signaling is central to GM-CSF action [9], we hypothesized that diminished GM-CSF action in the CNS may contribute to the obesity of *Stat5^fl/fl; Nestin-Cre^* animals. While the antibodies against phosphorylated STAT5 that we tested functioned poorly for immunohistochemistry in brain tissue, immunofluorescent detection of total STAT5 protein was robust and revealed the depletion of diffuse STAT5 staining and the increased intensity of STAT5 nuclear staining in the hypothalamus in response to i3vt GM-CSF injection in rats, consistent with GM-CSF-dependent activation and nuclear accumulation of STAT5 isoforms in the hypothalamus (Figure 9A). Indeed, counting of STAT5-immunoreactive nuclei in confocal images of these stained sections of rat brains revealed that i3vt GM-CSF administration doubled immunohistochemically-detectable STAT5 nuclear accumulation from approximately 20% to 40% of LHA neurons (Figure 9B). Thus, GM-CSF increases STAT5 nuclear accumulation in specific hypothalamic neurons, as in peripheral target tissues [20]. In order to determine a potential role for STAT5 in the anorexigenic actions of GM-CSF, we examined the response of control and *Stat5^fl/fl; Nestin-Cre^* mice to i3vt GM-CSF (Figure 9C). While i3vt administration of 1 µg of mouse GM-CSF resulted in a significant reduction of food intake as early as 4 hours in control *Stat5^fl/fl^* mice, there was no suppression of food intake in *Stat5^fl/fl; Nestin-Cre^* mice (Figure 9C; see description of two-way ANOVA test). In summary, these results suggest that the effects of GM-CSF to suppress food intake require CNS STAT5.

### Role for STAT5 in the regulation of the LHA

**Figure 10:**
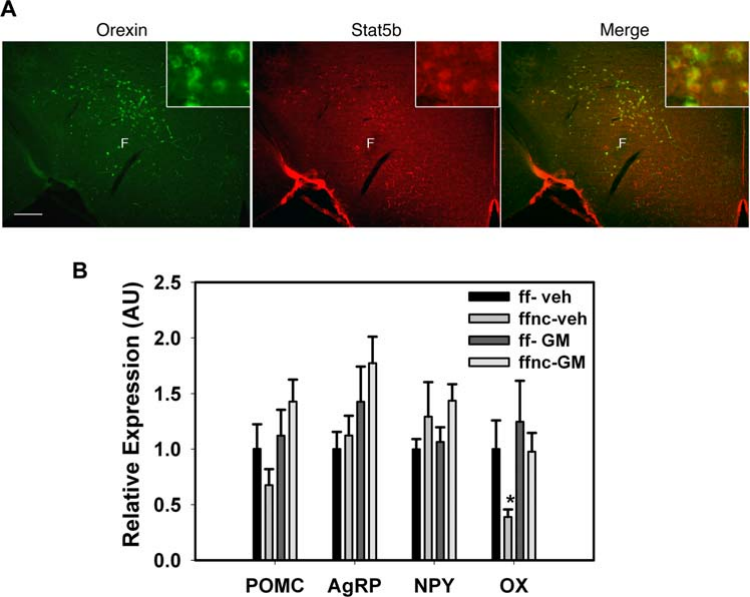
Potential role for Stat5 in OX-expressing LHA neurons in mice. (A) Histochemical sections from perfused wild-type male mice were subjected to immunostaining for OX (green, left) and STAT5B (red, middle). Right panel shows merged images demonstrating co-localization of prominent STAT5B expression with OX-expression in the LHA. Insets: magnified view of co-labeled neurons. F = fornix. (B) Total RNA prepared from whole hypothalamus tissue from and *Stat5^fl/f^* (ff) and *Stat5^fl/fl; Nestin-Cre^* (ffnc) mice treated with vehicle (veh) or GM-CSF (GM) was subjected to semi-quantitative real-time PCR for POMC, AgRP, NPY and OX mRNA expression. n>5; *p = 0.01 versus Wt Veh, S5, Veh by ANOVA F(3, 55) = 4.383 with Fisher's LSD test.

While our prior analysis of Arc mRNA expression did not reveal a role for STAT5 in the regulation of Arc neuropeptide gene expression, we noticed a population of highly STAT5-immunoreactive neurons dorsal to the fornix in LHA of wild-type animals (Figure 1A). Our subsequent analysis demonstrated this to be almost exclusively STAT5B (data not shown). Since our qPCR analysis also demonstrated the most complete reduction of STAT5 mRNA expression in the LHA, we examined the potential role for STAT5 in the LHA (Figure 10). We initially examined the co-localization of STAT5B in the LHA with the appetite- and activity-regulating neuropeptide, orexin (OX) by immunofluorescence (Figure 10A), revealing the virtually complete overlap of neurons demonstrating the prominent expression of STAT5B with those containing immunoreactive OX. These data suggest the potential importance of STAT5 in the regulation of these LHA OX neurons, prompting us to examine the regulation of Arc neuropeptide and *Hcrt* gene expression in RNA prepared from the hypothalami of the control and *Stat5^fl/fl; Nestin-Cre^* mice following i3vt GM-CSF treatment (Figure 10B). This analysis confirmed our previous finding that Arc neuropeptide mRNA expression is normal in *Stat5^fl/fl; Nestin-Cre^* mice, and also demonstrated the reduced expression of OX mRNA in the hypothalamus of vehicle-treated *Stat5^fl/fl; Nestin-Cre^* mice compared to controls. Acute i3vt GM-CSF treatment restored the hypothalamic expression of OX to normal levels in *Stat5^fl/fl; Nestin-Cre^* mice, however, suggesting that STAT5 mediates GM-CSF-induced anorexia by OX-independent mechanisms, and that GM-CSF can regulate OX mRNA expression independently of STAT5. Overall, however, STAT5B is highly expressed in LHA OX neurons and regulates baseline OX expression, suggesting that STAT5 in LHA OX neurons may be important for the regulation of energy balance.

## Discussion

**Figure 11:**
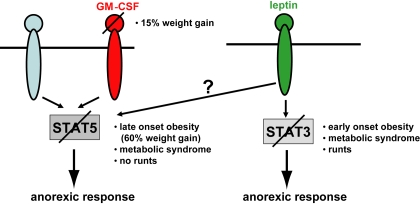
Summary diagram of cytokine signaling pathways controlling body weight. While leptin preferentially signals through STAT3, GM-CSF utilizes STAT5. However, since the GM-CSF-null mice develop milder symptoms than the STAT5-mutant mice described in this study, we propose that some of the STAT5 signaling is induced by leptin or yet another unknown pathway.

As shown in this study, deletion of STAT5 in the CNS of mice results in increased food intake, significant obesity by 17 weeks of age, and impairment of the anorexic response to GM-CSF administration. While a large body of evidence has pointed towards a key role for hypothalamic STAT3 in the regulation of energy balance, the current results suggest an important role for STAT5 in the regulation of feeding and body fat stores in response to endogenous signals (summary diagram Figure 11). Specifically, we observed a link between STAT5 and GM-CSF, which is known to play a role in the regulation of energy balance. Loss of STAT5 in the CNS results not only in increased food intake, but also alters the regulation of energy expenditure, as demonstrated by the reduced metabolic rate in males and reduced tolerance to prolonged cold exposure.

A variety of cytokines (leptin, CNTF, GM-CSF) contribute to the regulation of food intake and energy homeostasis by the CNS [8], [21], [22], [23]. The cellular/neural responses to cytokines are mediated by JAK kinases and prominently involve the actions of STAT transcription factors, such as STAT3 and STAT5. While leptin, IL-6, and CNTF heavily rely on STAT3 as their downstream mediator, GM-CSF preferentially activates STAT5 [9]. We have now provided evidence that STAT5 is activated by GM-CSF signaling in the CNS and that CNS STAT5 is crucial for the anorexic actions of GM-CSF; central administration of GM-CSF decreased energy intake in control mice but not in mice that had lost STAT5 in the CNS.

Interestingly, however, the metabolic defects observed upon deletion of neuronal STAT5 were larger than those observed in GM-CSF^−/− ^mice, suggesting that STAT5 may mediate the effects of other cytokine signals beyond GM-CSF (Figure 11). In particular, while loss of GM-CSF resulted in an approximately 15% weight gain at 22 weeks of age [8], STAT5 mutant mice displayed a 60% weight gain. Differences were also observed in the fat mass between the two mutant strains, and there was no sign of the depot-specific differences in fat accumulation in the *Stat5^fl/fl; Nestin-Cre^* mice as were observed in GM-CSF-deficient mice [8]. Thus, although direct comparisons of this sort between the present study and the previously studied GM-CSF^−/−^ animals must be made with some caution due to differences in strain background and animal facility, several lines of evidence suggest that STAT5 may function to regulate energy balance in response to additional cytokines besides GM-CSF. Indeed, STAT5 is activated by prolactin (Prl) [11] in ARC dopamine neurons [10], [12], and by GH in somatostatin neurons [13]. Furthermore, while STAT3 is crucial to the leptin response, leptin activates both STAT3 and STAT5 [24]. Similarly, while STAT3 is crucial to the physiologic leptin response, the pan-CNS deletion of STAT3 compared to animals null only for leptin receptor (ObRb) results in a more severe phenotype [2], [3].

Since disruption of the STAT3 binding site on the ObRb results in an obesity syndrome different in numerous aspects from that of complete ObRb deficiency [2], it is possible that STAT5 may participate in leptin action, as well. However, mice with a deletion of STAT5 in ObRb-containing neurons using an ObRb-Cre transgene [25] did not develop obesity over a period of weeks (Figure S1), suggesting that STAT5 controls energy balance through yet to be identified neurons. Since OX neurons do not express ObRb (data not shown), this finding is consistent with a potential for STAT5 in OX neurons in the regulation of energy balance.

Since the obesity observed in STAT5-mutant mice could be the result of altered GH or Prl levels, due to the absence of STAT5 in a subset of hypothalamic neurons, we used the sensitive measure of mammary gland development to gauge whether GH and Prl levels had been elevated in Stat5 mutant mice. Elevated levels of GH and Prl would result in a precocious development of mammary alveoli. No significant differences were observed in mammary alveolar development between control mice and mice from which the *Stat5* locus had been deleted using the Nestin-Cre or ObRb-Cre transgene (Figure S2), suggesting that these mice had not experience prolonged periods of eleveated GH and Prl levels.

STAT5 is expressed in different types of neurons and likely responds in a cell-specific fashion to a variety of cytokines to control many physiological responses. In a well-characterized neural circuit in the ARC, leptin activates anorexigenic (appetite-suppressing) neurons that express pro-opiomelanocortin (POMC) and inhibits the orexigenic (appetite-promoting) neurons that co-express neuropeptide Y (NPY) and Agouti-related protein (AgRP). While the exact neural pathway or pathways regulated by GM-CSF and/or STAT5 in the control of energy balance remain unknown, our analysis of ARC neuropeptide expression indicates that STAT5 operates outside of this circuit, since the expression of these neuropeptide mRNAs is not altered in *Stat5^fl/fl; Nestin-Cre^* mice. Since reduced SOCS3 expression in the Arc would tend to increase signaling by leptin and other cytokines to promote leanness, the regulation of hypothalamic SOCS3 expression by STAT5 is also unlikely to underlie the obesity of the *Stat5^fl/fl; Nestin-Cre^* mice.

The prominent expression of STAT5 in LHA OX neurons and the dysregulation of OX expression in the hypothalamus of *Stat5^fl/fl; Nestin-Cre^* mice suggests that STAT5 may operate in these neurons to regulate energy homeostasis. LHA OX neurons promote wakefulness and activity, and alteration of their function could underlie part of the decreased metabolism of the *Stat5^fl/fl; Nestin-Cre^* mice, although it is unlikely to account for the entire phenotype of these animals. We do not propose that STAT5 or GM-CSF constitute the only regulators of LHA OX neurons, nor do we suggest that OX is the only mediator of the metabolic action of Stat5. Indeed, the normalization of OX expression in the *Stat5^fl/fl; Nestin-Cre^* mice by GM-CSF treatment suggests that alterations in OX expression do not underlie the defects in GM-CSF action in the *Stat5^fl/fl; Nestin-Cre^* mice. Rather it is likely that STAT5 controls a multitude of genes involved in food intake and energy expenditure in the metabolically-important LHA OX neurons and elsewhere, and that the regulation of OX by STAT5 represents a single example of this.

STAT5 is also expressed and activated by cytokines in somatostatin neurons of the periventricular nucleus and dopamine neurons of the arcuate nucleus [13]. While the lean mass of *Stat5^fl/fl; Nestin-Cre^* mice was increased, neither GH, IGF-1 nor Prl levels were elevated in these animals, however. Therefore, the mechanism underlying the increased lean mass in the absence of neuronal STAT5 remains to be determined.

Consistent with the importance of cytokines in the regulation of body energy homeostasis, and with the central role of STAT proteins in the cellular response to cytokines, our results demonstrate a role for neural STAT5 in the regulation of food intake and energy utilization in response to endogenous signals. We furthermore demonstrate that STAT5 is critical to the anorexic response to GM-CSF, a known regulator of energy homeostasis. This novel role for STAT5 thus defines a key player in the CNS-mediated control of energy balance.

## Methods and Materials

### Generation of neuron-specific STAT5 knockout mice

*Stat5^fl/fl^* mice [26] were bred with a mouse containing the *Nestin-Cre* transgene [27]. *Stat5^fl/+; Nestin-Cre^* mice were mated with *Stat5^fl/fl^* to generate *Stat5^fl/fl; Nestin-Cre^* mice. All experiments were performed by using progenies of *Stat5^fl/fl^* mice crossed with *Stat5^fl/fl; Nestin-Cre^* mice (129xC57BL/6 background). All mice were maintained on a 12-h light, 12-h dark cycle and fed water an open formula NIH-specified diet for maintenance, growth and reproduction (NIH-07 rodent diet; http://www.zeiglerfeed.com/ratmouse.asp). The NIDDK Animal Care and Use Committee approved all procedures and studies were conducted in accordance with National Institutes of Health guidelines. Genotyping was performed by PCR amplification of tail DNA from each mouse at 3 weeks of age. The primers for genotyping of the *Stat5* floxed allele were primer 1 (5′-GAA AGC ATG AAA GGG TTG GAG-3′), primer 2 (5′-AGC AGC AAC CAG AGG ACT AC-3′) and primer 3 (5′-AAG TTA TCT CGA GTT AGT CAG G-3′). Primer pair 1 and 2 amplified a fragment of 450 bp from wild-type mice and a 200-bp fragment was detected by primer 2 and 3; the latter is located in the pLoxpneo vector, in mice heterozygous or homozygous for the *Stat5* floxed allele. Using PCR we also detected Nestin-Cre-mediated recombination of the floxed Stat5 allele in the pituitary gland (data not shown).

### Analysis of hypothalamic RNA

Hypothalami were isolated from age-matched mice between 10-12 AM and snap-frozen whole or microdissected into various subregions using a mouse brain matrix before snap-freezing. Total RNA was isolated using Trizol reagent. For determination of relative RNA concentration, total RNA was subjected to automated fluorescent RT-PCR on an ABI 7700. GAPDH control, STAT5A, STAT5B, orexin and SOCS3 primers and probes were also supplied by ABI. POMC, AgRP and NPY primers and probes were as previously described (1). Each predicted RT-PCR product spanned an intron/exon junction. Each RT-PCR reaction was determined to be in the linear range for quantitation by comparison to serial dilutions of input RNA.

### Immunofluorescent analysis of hypothalamus

Mice or rats (treated with GM-CSF or vehicle i3vt for 60 minutes, where indicated) were deeply anesthetized (90 mg/kg sodium pentobarbital) and perfused transcardially with formalin. Removal of the brain, post-fixation, cryoprotection, sectioning and immunofluorescence were as described [28]. In brief, free-floating tissue sections were blocked in donkey serum and then incubated with primary antibody before washing, incubation with fluorophore-conjugated secondary antibodies, and mounting. Antibodies against total STAT5 were obtained from Cell Signaling, Inc., antibodies against STAT5B were from Santa Cruz, αHu was from Invitrogen, and αOX was from Calbiochem. Images were then captured under fluorescent microscopy with a digital camera. For quantitation of STAT5 nuclear localization, confocal images were utilized to assess co-localization of STAT5-immunoreactivity with DAPI.

### Serum analysis and glucose/insulin tolerance test

Blood glucose levels were determined from the tail vein using a glucometer (Accu-Chek, Roche, Indianapolis, IN). Serum insulin and leptin levels were measured by radioimmunoassay (Linco Research Inc., St. Charles, MO). Serum FA and triglyceride levels were analyzed in fed mice using a commercial FA kit (Roche, Indianapolis, IN) and the GPO-Trinder kit (Sigma Chemical Co., St. Louis, MO), respectively. Insulin and glucose tolerance test were performed in fasted animals after i.p. injections of either insulin (0.75U/kg body weight) or glucose (2 g/kg body weight). Blood glucose values were measured immediately before and 15, 30 and 60 min after insulin injection, and before and 15, 30, 60, and 120 min after glucose injection. GH level was measured in Harbor-UCLA Med Center (National Hormone and Peptide Program) and IGF-1 level was measured in St. Joseph Hospital (Bangor, ME).

### Body composition

Body composition was measured in conscious mice using an NMR analyzer (Echo MRI 3-in-1, Houston, TX).

### Fat analysis

For determining the size of individual fat depots, animals were dissected and the wet mass of four intra-abdominal depots (parametrial, retroperitoneal, mesenteric, perirenal) and one subcutaneous depot (inguinal) were weighed. For determining adipocyte size, adipose tissue was immediately fixed in a 10% buffered formalin solution. Fixed tissue was embedded in paraffin, cut into 5 µm sections, mounted, and stained with hematoxylin and eosin. Stained slides were examined with a Nikon Eclipse E600 microscope equipped with a SPOT RT (Real Time) digital camera at bright field and 20X magnification. Digital images were taken for 3 non-overlapped microscope fields in each fat pad from the same animal. The measurement of cross-sectional area of adipocytes was determined as described previously [29]

### Food intake, metabolic rate, locomotor activity, and body temperature

Each mouse was housed individually and the amount of food was measured at day 0 using a regular scale. Two days later, the amount of food left in the feeding rack was measured, subtracted from the initial weight of food and divided by 2. The same experiment was repeated twice with the same set of mice at the same age. Metabolic rate was measured in fed mice 6 month of age by indirect calorimetry using the Oxymax system (Columbus Instruments, Columbus, OH). Data were collected for 24 h at room temperature (23°C) and for 24 h at thermoneutral temperature (30°C). Locomotor activity (total and ambulating) was measured using an infrared activity monitoring system (Columbus Instruments, Columbus, OH) while the mice were in the metabolic cages. Data are expressed as the average of 24 h and normalized to (total body weight)^0.75^. Body temperature measurements were taken between 1 and 3 pm or at midnight using a rectal thermometer (model TH-5; Braintree Scientific, Massachusetts).

### Statistical analysis

All results are reported as mean±SEM for equivalent groups. Statistical analyses were performed using SigmaStat software. One-way, two-way ANOVA, Two Way Repeated Measures ANOVA or t tests were used. Results are presented as ANOVA: F(n,n) = XX.X, p<0.0X; t-test t(n) = XX.X, p<0.0X where n shows the degree of freedom.

### Third cerebroventricular cannulation surgery

A permanent cannula directed toward the third cerebral ventricle was implanted in *Stat5^fl/fl; Nestin-Cre^* and *Stat5^fl/fl^* control mice for central injection of GM-CSF. Mice were anesthetized by intraperitoneal injection of avertin (0.2 ml/g). Scalp fur was shaved and mice were placed in a stereotaxic device (Kopf Instruments; Tajunga, CA) with lambda and bregma at the same vertical coordinate. Coordinates for cannula placement were 0.825 mm posterior to bregma and on the midline. A small skull window was outlined with a fine dremel bit and removed with forceps; the sagittal sinus was displaced laterally prior to lowering a 26-gauge stainless steel cannula (Plastics One; Roanoke, VA) such that the cannula tip was 4.8 mm below bregma. Dental acrylic was used to secure the cannula to the skull. Cannulas were sealed with fitted obturators from the same vendor. Cannula placement was verified after the mice had regained presurgical body weight by measuring food intake in response to an i3vt (intra-third ventricular) injection of NPY. Food was removed for 2hr in the middle of the light phase, and at the end of that interval, 1μl NPY [5μg/1μl] was injected. Mice consuming at least 1g of chow in the subsequent 1hr were included in the study.

### GM-CSF injection

On the day of injection, recombinant mouse GM-CSF (R&D Systems; Minneapolis, MN) was reconstituted at 1μg/μl in a vehicle solution of 0.05% mouse albumin/saline and kept on ice until time of injection. Mice were weighed and assigned to weight-matched groups, and food was removed from cages at about 5 hr prior to onset of dark. GM-CSF or vehicle was drawn up in microinjectors and PE50 tubing (Plastics One; Roanoke, VA) fitted to Hamilton syringes and 1μl was injected at about 4hr prior to the onset of dark. Food hoppers were weighed and placed in cages just prior to onset of the dark, and food intake was measured 4 hrs later. Mice were allowed to rest for 1 week with ad libitum chow access. The groups were then reversed, and mice received a second i3vt injection of the alternate treatment, with food intake and body weight monitored at 4 hrs as in the first trial. Mice consuming at least 1 g of food within 4 hrs were included in the analysis.

## Supporting Information

Figure S1 Body weight of 20 week-old *Stat5^fl/fl; ObRb-Cre^* mice and *Stat5^fl/fl^* littermate controls.
(5.69 MB TIF)

Figure S2 Wholemount analysis of mammary glands from 24 week-old female mice. Ductal development and side branching are normal in control (a), *Stat5^fl/fl; Nestin-Cre^*, (b) and *Stat5^fl/fl; ObRb-Cre^* (c) mature virgins.
(2.64 MB TIF)
