# Supplementary figures and images for: Loss of Cytokine-STAT5 Signaling in the CNS and Pituitary Gland Alters Energy Balance and Leads to Obesity
Source: PLoS One. 2008 Feb 20;3(2):e1639. doi: 10.1371/journal.pone.0001639 (PMC2237899; doi:10.1371/journal.pone.0001639)

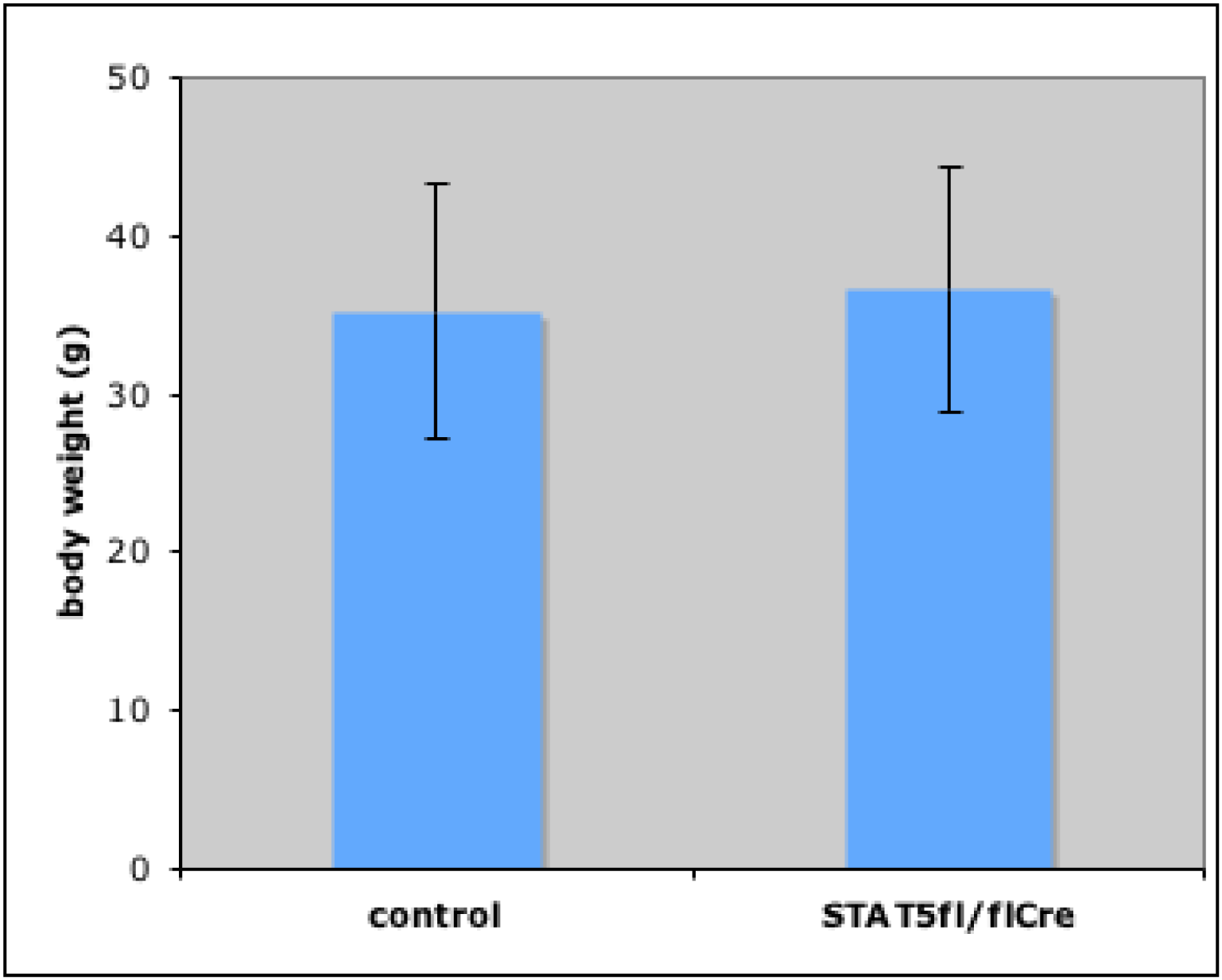

Supplement: Figure S1 — Body weight of 20 week-old Stat5fl/fl; ObRb-Cre mice and Stat5fl/fl littermate controls. (5.69 MB TIF) [file pone.0001639.s001.tif]

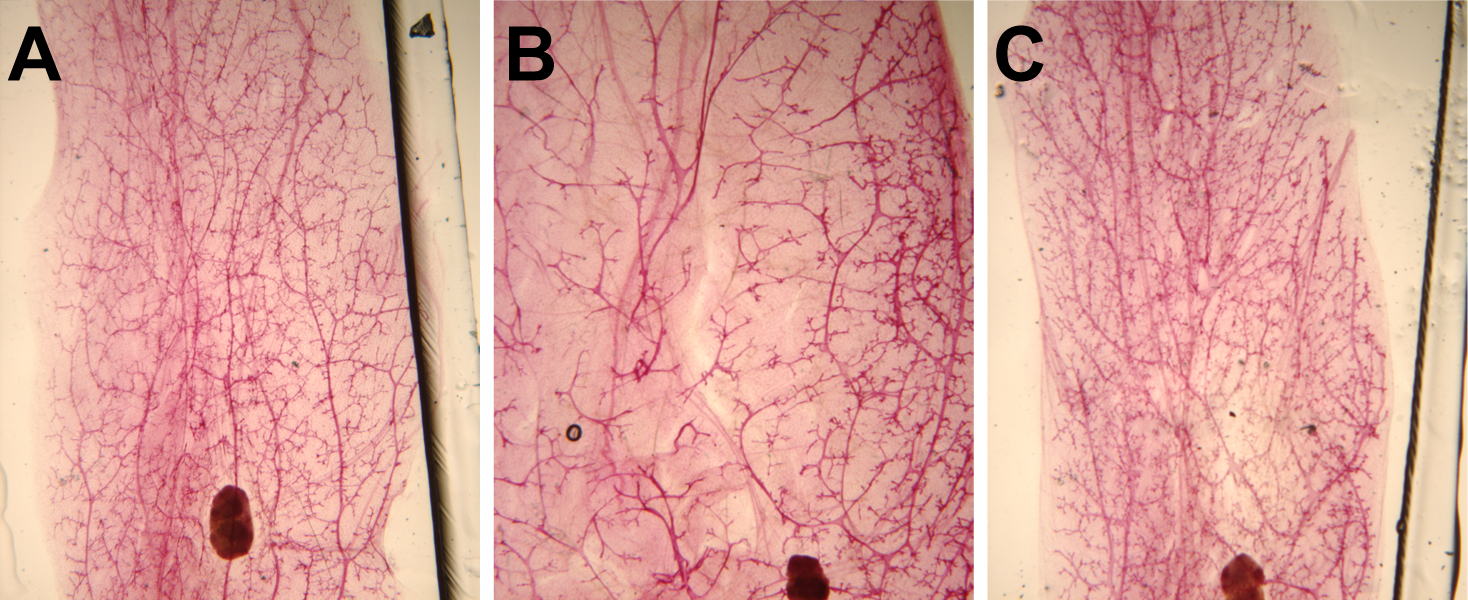

Supplement: Figure S2 — Wholemount analysis of mammary glands from 24 week-old female mice. Ductal development and side branching are normal in control (a), Stat5fl/fl; Nestin-Cre, (b) and Stat5fl/fl; ObRb-Cre (c) mature virgins. (2.64 MB TIF) [file pone.0001639.s002.tif]
